# Development of Radiolabeled Membrane Type-1 Matrix Metalloproteinase Activatable Cell Penetrating Peptide Imaging Probes

**DOI:** 10.3390/molecules200712076

**Published:** 2015-07-02

**Authors:** Sander M. J. van Duijnhoven, Marc S. Robillard, Klaas Nicolay, Holger Grüll

**Affiliations:** 1 Department of Biomedical Engineering, Eindhoven University of Technology, High Tech Campus 11, Eindhoven 5656 AE, The Netherlands; E-Mails: sander_van_duijnhoven@outlook.com (Sv.D.); k.nicolay@tue.nl (K.N.); 2 Department of Oncology Solutions, Philips Research, High Tech Campus 11, Eindhoven 5656 AE, The Netherlands; 3 Tagworks Pharmaceuticals, High Tech Campus 11, Eindhoven 5656 AE, The Netherlands; E-Mail: marc.robillard@tagworkspharma.com

**Keywords:** molecular imaging, activatable cell penetrating peptide, matrix metalloproteinase-14, membrane type-1 matrix metalloproteinase, radiolabeling, myocardial infarction, peptide chemistry

## Abstract

Membrane type-1 matrix metalloproteinase (MT1-MMP or MMP-14) plays an important role in adverse cardiac remodelling. Here, we aimed to develop radiolabeled activatable cell penetrating peptides (ACPP) sensitive to MT1-MMP for the detection of elevated MT1-MMP levels in adverse cardiac remodelling. Three ACPP analogs were synthesized and the most potent ACPP analog was selected using MT1-MMP sensitivity and enzyme specificity assays. This ACPP, called ACPP-B, showed high sensitivity towards MT1-MMP, soluble MMP-2, and MT2-MMP, while limited sensitivity was measured for other members of the MMP family. In *in vitro* cell assays, radiolabeled ACPP-B showed efficient cellular uptake upon activation. A pilot *in vivo* study showed increased uptake of the radiolabeled probe in regions of infarcted myocardium compared to remote myocardium, warranting further *in vivo* evaluation.

## 1. Introduction

The use of activatable cell penetrating imaging probes is a promising strategy for the *in vivo* detection of proteolytic activity in pathological conditions [1,2,3]. A radiolabeled activatable cell penetrating peptide (ACPP) sensitive toward matrix metalloproteinases (MMP)-2 and -9 was successfully employed for MMP detection in the course of remodeling post-myocardial infarction *in vivo* [1]. Besides activation in infarcted heart tissue, the MMP-2/9 sensitive ACPP probe also showed a considerable degree of activation in the vascular compartment, leading to background uptake of the activated probe in basically all tissues [1]. Therefore, the development of ACPPs which are only activated in the tissue of interest by tissue-specific enzymes should be the next step in optimization of the elegant ACPP concept. Membrane type-1 matrix metalloproteinase (MT1-MMP or MMP-14) has been identified to be such a tissue-specific target. MT1-MMP is involved in several protein modification and biological signaling pathways [4,5]. Specifically, MT1-MMP has been shown to convert the pro-enzyme MMP-2 into its active form, thereby facilitating extracellular matrix proteolysis [5,6]. Furthermore, MT1-MMP has been recognized as playing an important role in the profibrotic cascade by activation of the transforming growth factor-beta (TGF) pathway and subsequent induction of fibrillar collagens [5,6]. As such, MT1-MMP is involved in a variety of physiological, but also pathological tissue remodeling processes. For example in cancer, MT1-MMP plays a significant role in tumor growth and metastasis [7,8,9]. In atherosclerosis, MT1-MMP is directly linked to pathogenesis of plaque vulnerability [10]. Furthermore, MT1-MMP expression and activity is increased in pressure overload hypertrophy and in left-ventricular remodelling after cardiac ischemia-reperfusion injury or myocardial infarction, contributing to cardiac dysfunction [5,11,12,13,14]. In this respect, MT1-MMP upregulation holds great promise as an early biomarker for a variety of diseases. Several groups have reported on the development and/or application of MT1-MMP molecular imaging probes. MT1-MMP probes based on binding to the active site used peptide ligands or antibodies and were applied in near infrared fluorescence imaging, magnetic resonance imaging, or SPECT imaging [15,16,17,18]. Furthermore, MT1-MMP activatable molecular imaging probes have been developed for near infrared fluorescence imaging and SPECT imaging. Spinale and coworkers used an MT1-MMP specific fluorogenic substrate for MT1-MMP detection following cardiac ischemia-reperfusion injury [5,11]. Ouyang *et al*., developed a genetically encoded fluorescence resonance energy transfer (FRET) imaging probe by flanking the MT1-MMP cleavage domain in the pro-peptide sequence of MMP-2 by the fluorescent proteins ECFP and YPet [19]. This genetically encoded biosensor was further optimized by employing an MT1-MMP substrate, CRPAH-LRDSG, with enhanced sensitivity for MT1-MMP [20]. Watkins *et al*., investigated the use of an MT1-MMP ACPP for SPECT-imaging by inserting the MT1-MMP peptide substrate SGRIGF-LRTA between a radiolabeled polycationic and polyanionic peptide domain [21,22]. In this approach, *in vitro* activation was observed in cell cultures expressing MT1-MMP, but a control cell line lacking MT1-MMP expression also activated the probe, and therefore MT1-MMP selectivity could not be demonstrated. *In vivo* studies for this latter probe have not been reported. In a recent study, the similar substrate GRIGF-LRTA was demonstrated to possess high sensitivity for MT1-MMP, but also for MT2-MPP and MT3-MMP, while the sensitivity towards MMP-2 and MMP-9 was low [23]. By flanking the substrate with a Cy-5.5 fluorescent dye and a BHQ-3 quencher an organic FRET imaging probe was obtained, which was successfully used for the *in vivo* detection of membrane-type matrix metalloproteinase activity by optical imaging [23].

**Figure 1:**
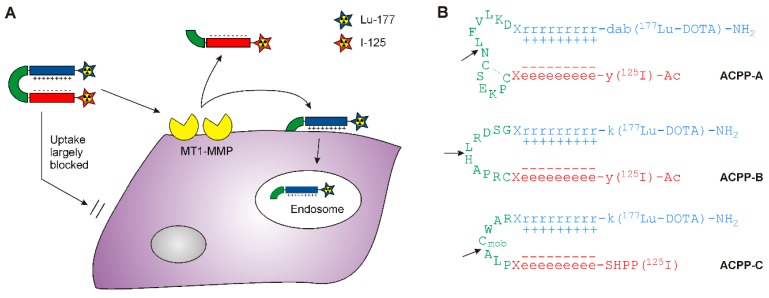
Mechanism and structure of proposed radiolabeled MT1-MMP activatable ACPPs. (**A**) The cell penetrating property of a polycationic peptide (in blue) is masked by a polyanionic peptide (in red). Cleavage of the substrate (in green) by MT1-MMP releases the polycationic cell penetrating peptide, which will transfer the radio-isotope ^177^Lu across the cell membrane, while the polyanionic domain together with the radioisotope ^125^I will be cleared from the tissue; (**B**) Peptide sequences of the three different MT1-MMP ACPP probes. The arrows indicate the MT1-MMP cleavage sites. In ACPP-A, the two cysteine residues form an intramolecular disulfide bridge. X and SHPP represent 3-oxapentanoic acid (O1Pen) and 3-(4-hydroxyphenyl)propionic acid, respectively. Design considerations for ACPP-C can be found in the supplementary information. The molecular structures of the different probes are presented in Figure S1.

Here, we aimed to develop novel radiolabeled MT1-MMP ACPPs by using three MT1-MMP substrates different from the study of Watkins and colleagues (Figure 1). These substrates include the MT1-MMP cleavage domain in the pro-peptide sequence of MMP-2, CPKESCN-LFVLKD and the more MT1-MMP sensitive CRPAH-LRDSG substrate [19,20]. Furthermore, the peptide PLA-C_mob_WAR, which has been successfully used in an organic FRET probe for the monitoring of MT1-MMP in heart disease, was employed as MT1-MMP-selective sequence [12]. The polycationic and polyanionic domains consisted of D-amino acids to avoid proteolytic degradation of these domains. The cell penetrating peptide domain of the ACPPs was functionalized with a DOTA chelate to enable radiolabeling with ^177^Lu. A dual-isotope radiolabeled MMP-2/9 ACPP (previously named dACPP, in this paper referred to as ACPP-2/9) approach recently proved valuable in the discrimination between intact and activated probe in *in vivo* biodistribution studies in mouse models of cancer and myocardial infarction [1,24,25]. Accordingly, we set out to develop dual-isotope radiolabeled MT1-MMP sensitive ACPPs using the orthogonal radio-isotopes ^177^Lu and ^125^I (Figure 1). To introduce the radiolabel ^125^I in the polyanionic inhibitory domain, either a tyrosine was introduced in the polyanionic inhibitory domain for direct radiolabeling, or ^125^I was introduced specifically to the N-terminus via coupling of a ^125^I labelled 3-(4-hydroxyphenyl)propionic acid (SHPP) residue. The probes (ACPP-A, -B, and -C) were synthesized and studied for their MT1-MMP sensitivity and specificity. The best performing probe was subsequently studied *in vivo* in a pilot study in a mouse model of myocardial infarction and compared to ACPP-2/9.

## 2. Results and Discussion

### 2.1. Probe Synthesis

**Figure 2:**
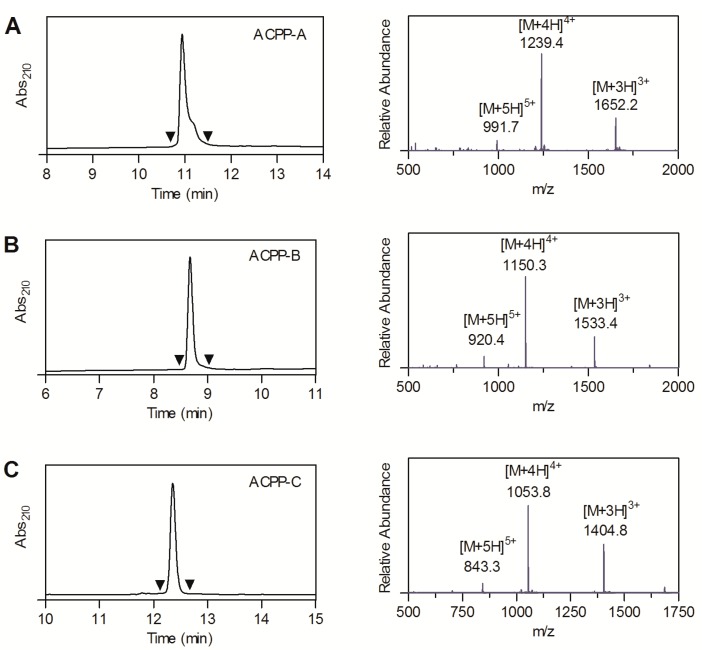
LC-MS characterization of (**A**) Ac-y-e_9_-X-C*PKESC*NLFVLKD-X-r_9_-dab(DOTA)-NH_2_ (ACPP-A, obsd. 4951.4 Da, calcd. 4951.4 Da); (**B**) Ac-y-e_9_-X-CRPAHLRDSG-X-r_9_-k(DOTA)-NH_2 _(ACPP-B, obsd. 4597.3 Da, calcd. 4597.3 Da), and (**C**) SHPP-e_9_-X-PLAC_mob_WAR-X-r_8_-k(DOTA)-NH_2_ (ACPP-C, obsd. 4209.1 Da, calcd. 4209.1 Da). C* represents cysteine residues that are linked via an intramolecular disulfide bridge. The left and right graphs show the UV absorbance chromatogram and the mass spectrum of the UV-peak bracketed by the arrowheads, respectively.

The peptides ACPP-A, ACPP-B, and ACPP-C with and without SHPP functionality were successfully prepared by Fmoc solid-phase peptide synthesis and in-solution coupling strategies, and purified by reversed phase—HPLC. Liquid chromatography—mass spectrometry (LC-MS) analysis demonstrated >95% pure peptides with molecular masses consistent with their theoretic masses (Figure 2).

### 2.2. MT1-MMP Sensitivity of ACPP Probes

**Figure 3:**
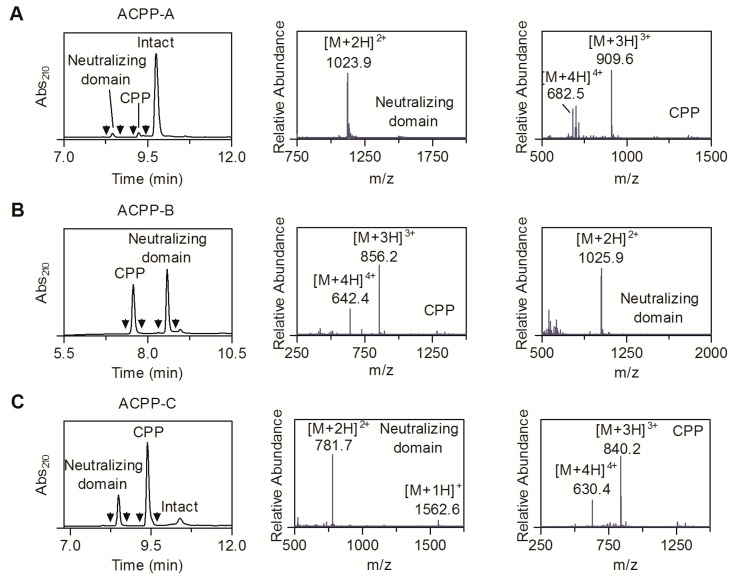
LC-MS characterization of ACPP-A, ACPP-B, and ACPP-C (0.1 mM) incubated with MT1-MMP (0.13 µM) for 60 min. The left and right graphs show the UV absorbance chromatogram and the mass spectra of the UV-peaks bracketed by the arrowheads, respectively. (**A**) ACPP-A; MS spectra of Neutralizing domain (obsd. 2244.8 Da, calcd. 2244.8 Da for Ac-y-e_9_-X-C*PKESC*N-COOH), and CPP (obsd. 2724.7 Da, calcd. 2724.7 Da for H_2_N-LFVLKD-X-r_9_-dab(DOTA)-NH_2_). (**B**) ACPP-B; MS spectra of CPP (obsd. 2565.5 Da, calcd. 2565.5 Da for H_2_N-LRDSG-X-r_9_-k(DOTA)-NH_2_), and Neutralizing domain (obsd. 2049.8 Da, calcd. 2049.8 Da for Ac-y-e_9_-X-CRPAH-COOH). (**C**) ACPP-C (without SHPP functionality); MS spectra of Neutralizing domain (obsd. 1561.5 Da, calcd. 1561.5 Da for e_9_-x-PLA-COOH), and CPP (obsd. 2517.4 Da, calcd. 2517.4 Da for H_2_N-C_mob_WAR-X-r_8_-k(DOTA)-NH_2_). C* represents cysteine residues that are linked via an intramolecular disulfide bridge.

The peptides ACPP-A, ACPP-B, and ACPP-C (without SHPP functionality) were screened for MT1-MMP sensitivity by incubating the peptides (0.1 mM) with MT1-MMP (0.13 µM) for 60 min. For ACPP-A, a small fraction (<5%) of the peptide was cleaved between the asparagine and leucine residue (Figure 3A). In contrast, ACPP-B and ACPP-C were efficiently cleaved by MT1-MMP (Figure 3B,C). ACPP-A analogs (a peptide probe lacking the 5-amino-3-oxapentanoic acid linker residues and its linear analog) were also synthesized and tested for MT1-MMP sensitivity, showing also a low degree of activation (Figure S2). Accordingly, a recent study found a poor response of a genetically encoded MT1-MMP biosensor employing the same substrate [20]. The low sensitivity of ACPP-A for MT1-MMP and the fact that ACPPs are expected to be cleared from the circulation relatively fast (blood half-life times of <30 min) [24], does not hold much promise for significant accumulation of ACPP-A in MT1-MMP expressing tissue, precluding *in vivo* imaging of MT1-MMP with this particular probe. Therefore, further in-depth analysis of the MT1-MMP specificity and *in vivo* characteristics of ACPP-A was not performed.

### 2.3. Enzyme Specificity of ACPP-B & ACPP-C

**Figure 4:**
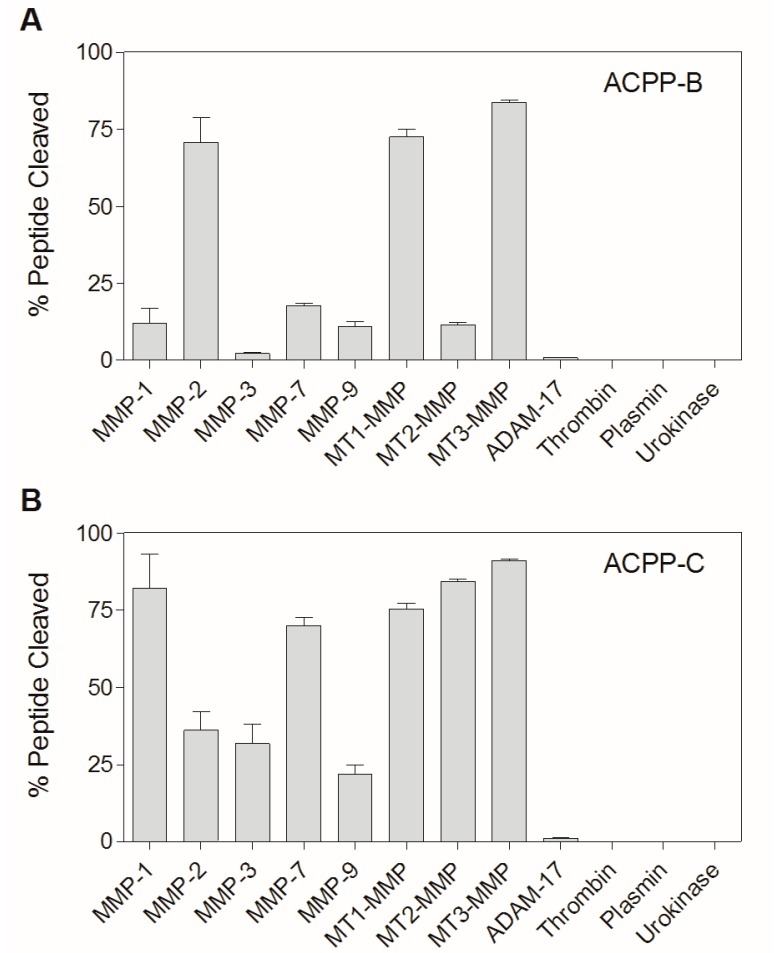
The percentage of released CPP from (**A**) ACPP-B and (**B**) ACPP-C (0.1 mM) after 1 h incubation with 12 different enzymes (20 nM) in vitro. The experiments were performed in triplicate and the data are presented as mean ± SD. Note: ACPP-C was incubated with 67 nM MMP-7 instead of 20 nM.

ACPP-B and ACPP-C were incubated with twelve different enzymes, including the soluble matrix metalloproteinases MMP-1, MMP-2, MMP-3, MMP-7, and MMP-9, and the catalytic domains of MT1-MMP, MT2-MMP, and MT3-MMP (Figure 4). The sensitivity of the probes was also tested for the catalytic domain of ADAM-17, a desintegrin and metalloproteinase that is upregulated in heart failure. Finally, the sensitivity was determined towards enzymes present in the circulation, *i.e*., urokinase, plasmin, and thrombin. LC-MS analysis after 1 h of incubation showed that ACPP-B is efficiently cleaved between the histidine and leucine residue by MMP-2, MT1-MMP, and MT3-MMP. The other members of the MMP family effected minor degradation of ACPP-B, while negligible cleavage was observed for ADAM-17 and no cleavage was observed for thrombin, plasmin, and urokinase (Figure 4A).

ACPP-C was efficiently cleaved, between the alanine and C_mob_ residue, by MMP-1, -7, MT1-MMP, MT2-MMP, and MT3-MMP, and to a modest extent by MMP-2, -3, and -9, while no ACPP-C cleavage was observed for ADAM-17, thrombin, plasmin, and urokinase (Figure 4B). Because MT1-MMP specificity was reported for the substrate PLAC_mob_WAR flanked by two fluorophores, we were surprised to observe efficient cleavage of ACPP-C by other MMPs [11]. Attachment of the relatively large and charged peptide domains may have adversely affected the cleavage rate for MT1-MMP in competition with other MMPs. Due to the broad MMP sensitivity for both tissue-specific and soluble MMPs, ACPP-C is most likely also activated in the vascular compartment, and, as a result, represents at best only a marginal improvement compared to the ACPP-2/9 with respect to the specific targeting of proteolytic activity involved in myocardial remodeling [1]. ACPP-B was selected for further *in vitro* and *in vivo* analysis as it showed the most MT-MMP specificity.

### 2.4. In Vitro Cell Assay ACPP-B

**Figure 5:**
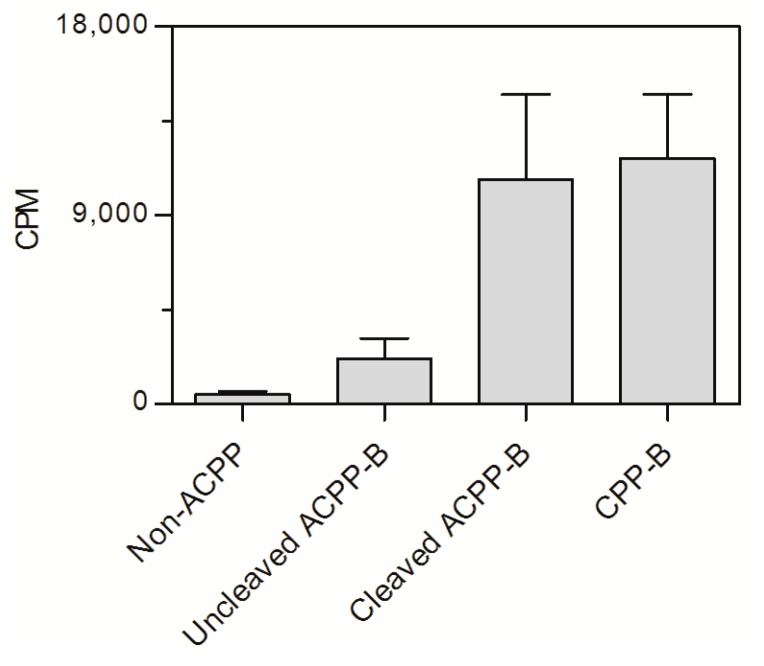
Cellular uptake of ^177^Lu-non-ACPP, uncleaved ^177^Lu-ACPP-B, pre-activated ^177^Lu-ACPP-B, and ^177^Lu-CPP-B after 3h incubation with HT-1080 cells, assessed by γ-counting. Data are presented as mean ± SD.

Next, we radiolabeled the polycationic domain of ACPP-B with ^177^Lu (Figure S3) and set out to assess the uptake of ^177^Lu-ACPP-B by MMP-positive HT-1080 cells. Although these cells secrete MMPs, including MT1-MMP [26], it has been demonstrated that absolute enzyme levels are relatively low in 2-dimensional cell culture experiments, resulting in minimal ACPP probe activation and insignificant uptake [27]. Therefore, we employed the same approach as previously reported for ACPP-2/9 [24,27]. That means, the cellular uptake was compared between radiolabeled pre-cleaved ^177^Lu-ACPP-B (>95% cleavage, Figure S3), uncleaved ^177^Lu-ACPP-B, a negative control and a positive control. ^177^Lu-non-ACPP [24], which showed no MT1-MMP sensitivity, and ^177^Lu-CPP-B served as negative and positive control, respectively. CPP-B was obtained by MT1-MMP-mediated cleavage of ACPP-B and subsequent isolation of the polycationic domain by reversed phase-HPLC. After 3 h incubation at 1.25 µM, the cellular uptake of the activated ^177^Lu-ACPP-B was comparable to uptake of the positive control ^177^Lu-CPP-B, and furthermore significantly higher with respect to uncleaved ^177^Lu-ACPP-B, and ^177^Lu-non-ACPP (Figure 5), demonstrating efficient silencing of the CPP activity before probe activation. The concentration of 1.25 µM was four-fold lower compared to the intermolecular K_m_ between the polyanionic and polycationic domain [27]. As such, pre-cleavage of ACPP-B was expected to result in efficient dissociation of the polyanionic and polycationic domain, leaving the activated CPP free for cellular adhesion and subsequent uptake.

Although not significant, intact ^177^Lu-ACPP-B exhibited a slightly higher uptake and retention compared to the negative control ^177^Lu-non-ACPP (Figure 5), while this was not observed for a MMP-2/9 sensitive ^177^Lu-ACPP [24]. We continued to investigate if the modest uptake of intact ACPP-B was possibly mediated through activation by membrane-type MT1-MMP and MT3-MMP expressed by HT-1080 cells [26]. Thereto, the negative control, intact ACPP-B, and pre-cleaved ACPP-B were incubated with HT-1080 cells in the presence of a broad-spectrum MMP inhibitor, GM6001. The presence of GM6001 did not affect the cellular uptake of ACPP-B (Figure S4), and this suggests that MT-MMP mediated activation did not play a role and that the difference in cellular uptake between ACPP-B and the negative control is most likely caused by differences in probe structure. In these experiments, we used the scrambled negative control for the MMP-2/9 ACPP [24] that also showed no MT1-MMP sensitivity. However, the linkers for ACPP-B and non-ACPP, O1Pen-CRPAHLRDSG-O1Pen and Ahx-LALGPG respectively, are rather different. A major dissimilarity is that the linker of non-ACPP consists solely of hydrophobic, non-polar amino acids, while the linker of ACPP-B mainly contains polar amino acids. It might be that the linker of ACPP-B can interact with proteins/cells via hydrogen bonding mediating some cellular retention. Another difference is that the linker of ACPP-B consists of 42 bonds *versus* 25 bonds for the linker in non-ACPP. Therefore, the effective molarity (EM) [27,28] for ACPP-B, 23 mM, is lower compared to non-ACPP, 49 mM, and may lead to a higher level of dissociation of the polycationic and polyanionic domain and thus less efficient masking of the cell penetrating property of the polycationic peptide in ACPP-B. Yet, efficient intramolecular hairpin formation and subsequent masking of the cell penetrating property is expected for both probes as the EMs are much higher than the 5.9 µM intermolecular affinity between the polycationic and polyanionic domain [27].

### 2.5. In Vivo Biodistribution

An *in vivo* pilot study was performed to assess the *in vivo* biodistribution of ACPP-B and CPP-B, 5 h post-injection, in a mouse model of myocardial infarction (MI). The conditions were similar to the earlier study with the MMP-2/9 sensitive probe ^177^Lu/^125^I-ACPP-2/9 [1] to facilitate comparison. We aimed to characterize the *in vivo* biodistribution of ACPP-B using a dual-isotope approach with ^177^Lu, labeled to the polycationic domain, and ^125^I, labeled to the polyanionic domain, as orthogonal radiolabels (see Supplemental Information for performed iodination procedures). Although iodination of ACPP-B resulted in efficient conjugation of ^125^I to the peptide (Figure S5), the oxidative conditions, needed for activation of ^125^I, resulted in disulfide formation between cysteine residues, resulting in ACPP-B peptide dimers (Figure S6). An alternative approach, activating ^125^I in iodination tubes prior to conjugation to ACPP-B in a normal tube resulted in ~60% labeling. Unfortunately, a >95% radiochemical purity could not be achieved by solid phase extraction (Figure S5), and desalting strategies to remove ^125^I could not be applied due to the low molecular weight of the peptide. Therefore, re-design of the ACPP-B peptide structure, to prevent side-reactions during iodination, may enable dual-isotope studies. Hereto, the cysteine residue can be acetylated, but oxidation of the sulfur atom may still occur. Furthermore, this conversion results in a more hydrophobic residue that may impact the MT1-MMP sensitivity of the probe. Substitution of the cysteine by a serine residue will possibly be a more attractive option. Alternatively, iodine may be introduced specifically to the N-terminus via coupling of a ^125^I-SHPP moiety (following a similar approach as proposed for iodination of ACPP-C (Figure 1)).

**Figure 6:**
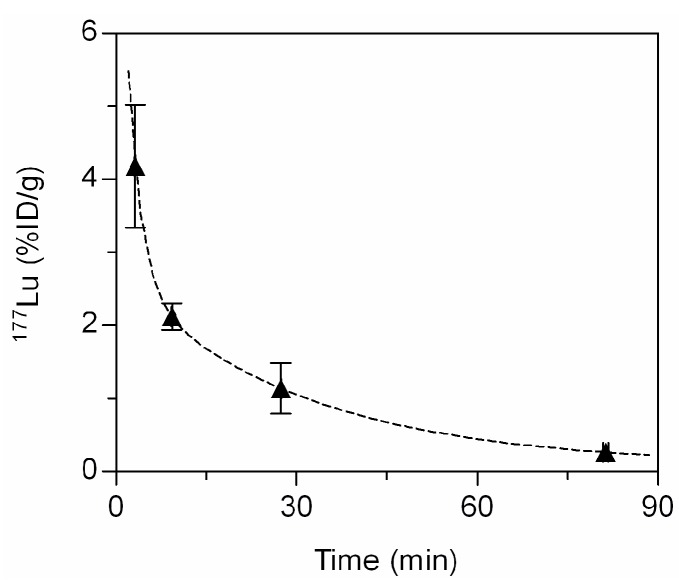
Blood kinetics of ^177^Lu-ACPP-B in MI-mice (*n *= 3). The data are mean %ID/g ± SD and fitted to the 2-phase exponential decay function Y = span_1_ × exp(−K_1_ × X) + span_2_ × exp(−K_2_ × X) + Plateau. Span_1_ = 2.61%ID/g, K_1_ = 1.99 min^−1^, Span_2_ = 7.35%ID/g, K_2_ = 27.1 min^−1^.

Here, a single isotope labeled probe ^177^Lu-ACPP-B was employed in a pilot *in vivo* study. The biodistribution studies in MI-mice, 10 days after induction of a myocardial infarction, revealed a significantly higher uptake of ^177^Lu radiolabeled ACPP-B (*p *< 0.001) in infarcted regions of the heart compared to remote myocardium (Table 1). Evaluation of ACPP-2/9 in the same mouse model earlier demonstrated a higher uptake of ACPP-2/9 in the infarcted heart compared to ACPP-B, but also in other tissues such as remote myocardium, muscle, lung, spleen, and liver, while the accumulation of ACPP-2/9 in kidney was lower compared to ACPP-B (Table 1). Furthermore, the studies exploring ACPP-2/9, non-ACPP-2/9, and CPP-2/9, revealed that the activated domain of the ACPP-2/9 imaging probe is efficiently taken up by the liver, while the intact probe is accumulated in the kidneys [1,24,25]. These experiments showed that a significant fraction of ACPP-2/9 was activated in the vasculature and subsequently taken up by the liver with a kidney-to-liver ratio of 2.1 (Table 1). Importantly, ACPP-B mainly accumulated in the kidneys with a kidney-to-liver ratio of 14.9, and this suggests a diminished activation of ACPP-B in the vasculature. Notably, ACPP-B showed a similar blood kinetic profile (Figure 6) as ACPP-2/9 [1], with a blood half-life of ~24 min. The calculated volume-of-distribution for ACPP-B is 0.3 L/kg, which is also in the same range as for ACPP-2/9, and indicates that the probe is rapidly distributed throughout the extracellular extravascular space [29]. These results indicate that the differences between ACPP-2/9 and ACPP-B activation in the vasculature cannot be attributed to difference in blood kinetics, but are most likely the result of the difference in sensitivity for circulating MMPs. We demonstrated that ACPP-2/9 is sensitive towards the soluble proteases MMP-2 and MMP-9 [24], while ACPP-B showed *in vitro* sensitivity towards MMP-2, but not for MMP-9 (Figure 4).

**Table 1:** Biodistribution results of 10 nmol ^177^Lu-ACPP-B, and ^177^Lu-ACPP-2/9 5 h post-injection in MI-mice. The data are mean %ID/g ± SD. Data for ^177^Lu-ACPP-2/9 are reprinted from [1].

	^177^Lu-ACPP-B ( *n* = 5)	^177^Lu-ACPP-2/9 ( *n *= 3)
Blood	0.08 ± 0.03	0.09 ± 0.03
Heart, infarct	0.75 ± 0.15	3.60 ± 0.68
Heart, remote	0.11 ± 0.02	0.35 ± 0.13
Muscle	0.09 ± 0.07	0.48 ± 0.14
Lung	0.38 ± 0.09	1.09 ± 0.30
Spleen	0.77 ± 0.13	2.72 ± 1.48
Liver	6.76 ± 0.91	28.5 ± 5.33
Kidney	100.4 ± 13.0	59.9 ± 8.10
Fat	0.43 ± 0.28	0.33 ± 0.08
Thigh bone	1.92 ± 0.73	2.47 ± 1.02
Brain	0.01 ± 0.01	0.02 ± 0.01

**Figure 7:**
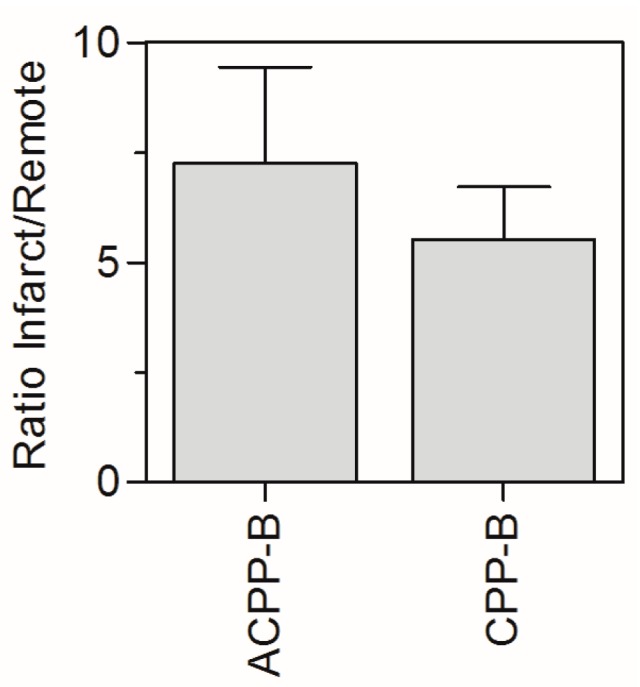
Infarct-to-remote ratio for ^177^Lu-ACPP-B and ^177^Lu-CPP-B. The data are mean ± SD.

Acute MI is characterized by an increased vascular permeability and increased interstitial space [30]. Therefore, the increased uptake of ^177^Lu-ACPP-B in the infarcted region compared to remote region does not necessarily warrant MT1-MMP mediated probe activation but can also be associated with non-specific leakage of the ACPP-B probe into the infarcted myocardial interstitial space. We therefore compared the infarct-to-remote ratio for ^177^Lu-ACPP-B and the positive control ^177^Lu-CPP-B (Supplemental Table 1) and found an increased ratio for ACPP-B (Figure 7), suggesting that infarct uptake of ^177^Lu-ACPP-B is associated with tissue-specific activation. However, the difference was not statistically significant (*p* = 0.17) due to the relatively high variation for ACPP-B. The high variation is most likely related to the relatively high biological variation typically observed for the MI-model [31]. This MI-mouse model is known for its relatively high mortality and more importantly large variation in myocardial ischemia and infarct size. We therefore believe that high variation in biodistribution data is caused by the relatively high biological variation (e.g., MMP expression) per MI-mouse as a result of the variability in the surgical intervention. Extended biodistribution studies and, in particular, dual-isotope studies would be valuable to verify if ACPP-B probe activation occurs *in vivo* and to specify the localization, with a special interest in infarcted and remote areas of the heart. Such studies could further address the question if ACPP-B will out-perform ACPP-2/9 in terms of specific targeting of infarcted myocardium.

## 3. Experimental Section

### 3.1. Materials

All reagents and solvents were obtained from commercial sources (Sigma-Aldrich, St. Louis, MO, USA, and Biosolve, Lexington, MA, USA) and used without further purification. 9-fluorenylmethyloxycarbonyl (Fmoc)-protected amino acids and Rink amide resin were purchased from either Novabiochem (Merck, Kenilworth, NJ, USA) or Iris Biotech. DOTA succinimidyl ester was obtained from Macrocyclics. Human recombinant enzymes were purchased from Calbiochem (Merck), Sigma-Aldrich, or R&D Systems (Minneapolis, MN, USA). The enzymes were activated according to vendors’ protocols if needed.

### 3.2. Probe Synthesis

Peptides Ac-y-e_9_-X-CPKESCNLFVLKD-X-r_9_-dab(ivDde)-resin, Ac-y-e_9_-X-CRPAHLRDSG-X-r_9_-k(ivDde)-resin, and Fmoc-e_9_-X-PLAC_mob_WAR-X-r_8_-k(Mtt)-resin were synthesized on an automatic synthesizer (Prelude, Protein Technologies Inc., Tucson, AZ, USA ) by Fmoc solid-phase peptide synthesis (SPPS) using Chemmatrix rink amide resin (0.1 mmol), HBTU as the activating reagent, and *N*, *N*-Diisopropylethyl amine (DIPEA) as base. d-amino acids are denoted in lower case. X and C_mob_ represents 5-amino-3-oxapentanoic acid and 4-methoxybenzyl-l-cysteine, respectively. The ivDde protecting group was selectively removed by incubating Ac-y-e_9_-X-CPKESCNLFVLKD-X-r_9_-dab(ivDde)-resin, and Ac-y-e_9_-X-CRPAHLRDSG-X-r_9_-k(ivDde)-resin with 5.0% *v/v* hydrazine-monohydrate in DMF for 10 × 3 min. The Mtt protecting group was selectively removed by incubating Fmoc-e_9_-X-PLAC_mob_WAR-X-r_8_-k(Mtt)-resin with 1.8% *v/v* trifluoroacetic acid (TFA) in dichloromethane with 2.0% *v/v* tri-isopropylsilane (TIS) as scavenger for 10 × 3 min. DOTA succinimidyl ester (4.0 equiv) in NMP was added to the peptide resins and was reacted for 1 h in the presence of DIPEA. After Fmoc removal, the peptides were cleaved from the resin by a mixture of 90.5% *v/v* trifluoroacetic acid (TFA), 2.0% *v/v* 1,2-ethanediol (EDT), 5.0% *v/v* tri-isopropyl silane (TIS), and 2.5% *v/v* MilliQ (Millipore, Billerica, MA, USA) water for 4 h, filtered, and precipitated in ice cold diethylether. The peptide pellets were dissolved in MilliQ water and purified by preparative reversed-phase high pressure liquid chromatography (RP-HPLC) using an Agilent 1200 apparatus, equipped with a C18 Zorbax column (150 × 21.2 mm, particle size 5.0 µm). The elution gradient was set from 5% to 30% of buffer B (0.1% TFA in acetonitrile) over 100 min, where buffer A was 0.1% TFA in MilliQ (Millipore) water. The UV wavelength was preset at 210 and 254 nm. All peptides structures were analyzed by LC-MS on an Agilent 1200 apparatus, equipped with a C18 Eclipse plus-column (100 × 2.1 mm, particle size 3.5 µm) and an electrospray mass spectrometer (Agilent Technologies 6210, Time-of-Flight LC/MS, Santa Clara, CA, USA) Peptide Ac-y-e_9_-X-CPKESCNLFVLKD-X-r_9_-dab(DOTA)-NH_2_ was cyclized at 50 rpm in 15% *v/v* DMSO, 10% *v/v* acetonitrile, and 75% *v/v* MilliQ water for 3 days, purified by RP-HPLC, and analyzed by LC-MS. The positive control of Ac-y-e_9_-X-CRPAHLRDSG-X-r_9_-k(DOTA)-NH_2_ was obtained by incubating 50% of this peptide (1.0 mM) with 60 nM MT1-MMP in a mixture of 50 mM Tris, 200 mM NaCl, 10 mM CaCl_2_ and 10 µM ZnCl_2_ at pH 7.5 at 700 rpm and 37 °C for 24 h, after which the polycationic domain was isolated by RP-HPLC, and analyzed by LC-MS. To test SHPP introduction, 50% of peptide e_9_-X-PLAC_mob_WAR-X-r_8_-k(DOTA)-NH_2_ was reacted with 3-(4-hydroxyphenyl)propionic acid (SHPP) *N*-hydroxysuccinimide ester (10 equiv) in DMF in the presence of DIPEA for 6 h, after which the peptide was purified by RP-HPLC, and analyzed by LC-MS.

Found masses: 4953.4 Da for Ac-y-e_9_-X-CPKESCNLFVLKD-X-r_9_-dab(DOTA)-NH_2_ (Calcd. 4953.4 Da), 4951.4 Da for Ac-y-e_9_-X-C*PKESC*NLFVLKD-X-r_9_-dab(DOTA)-NH_2_ (ACPP-A, Calcd. 4951.4 Da), 4597.3 Da for Ac-y-e_9_-X-CRPAHLRDSG-X-r_9_-k(DOTA)-NH_2_ (ACPP-B, Calcd. 4597.3 Da), 2565.5 Da for LRDSG-X-r_9_-k(DOTA)-NH_2 _(CPP-B, Calcd. 2565.5 Da), 4061.2 Da for e_9_-X-PLAC_mob_WAR-X-r_8_-k(DOTA)-NH_2_ (Calcd. 4061.1 Da), and 4209.1 Da for SHPP-e_9_-X-PLAC_mob_WAR-X-r_8_-k(DOTA)-NH_2_ (ACPP-C, Calcd. 4209.1 Da).

### 3.3. MT1-MMP Sensitivity

ACPP-A, ACPP-B, and ACPP-C (0.1 mM) were incubated with 15 nM MT1-MMP in a mixture of 50 mM Tris, 200 mM NaCl, 10 mM CaCl_2_ and 10 µM ZnCl_2_ at pH 7.5 at 700 rpm and 37 °C. After 1 h, the enzyme was quenched by the addition of TFA (10% *v/v*), and the reaction mixture was analyzed by LC-MS.

### 3.4. Enzyme Assay

ACPP-B and ACPP-C were incubated in triplicate with 20 nM of either human recombinant MMP-1, MMP-2, MMP-3, MMP-7, MMP-9, MT1-MMP, MT2-MMP, MT3-MMP, ADAM-17, thrombin, plasmin, or urokinase in a mixture of 50 mM Tris, 200 mM NaCl, 10 mM CaCl_2_ and 10 µM ZnCl_2_ at pH 7.5 at 700 rpm and 37 °C. After 1 h, the enzymes were quenched by the addition of TFA (10% *v/v*) and the reaction mixtures were analyzed by LC-MS. The formation of the activated cell penetrating peptide domain was monitored and compared to a 100% cleaved reference sample.

#### 3.4.1. Radiolabeling

*For in vitro cell assay: *^177^LuCl_3_ (PerkinElmer, Waltham, MA, USA) in 0.05 M HCl (2.0 µL, 4.0 MBq) was mixed with ACPP-B, non-ACPP, CPP-B, or CPP in MilliQ water (2.69–4.60 µL, 20.0 nmol), and metal-free 0.9% NaCl (final volume 100 µL) for 20 min, at 600 rpm and 90 °C.

*For in vivo studies: *^177^LuCl_3_ in 0.05 M HCl (6.0 µL, 18.0 MBq) was mixed with ACPP-B or CPP-B in MilliQ water (10.4 µL, 45 nmol), and metal-free 0.9% NaCl (433.6 µL) for 20 min, at 600 rpm and 90 °C. The ^177^Lu labeling yields were determined by radio-TLC, using iTLC-SG strips (Pall, Port Washington, NY, USA) eluted with 200 mM EDTA in 0.9% NaCl, imaged on a phosphor imager (FLA-7000, Fujifilm, Tokyo, Japan) and quantified with AIDA Image Analyzer software. Analytical radio-HPLC was carried out on an Agilent 1100 system equipped with a C18 Eclipse XBD-column (150 × 4.6 mm, particle size 5 µm) and a Gabi radioactive detector (Raytest). The radiochemical purities were 95% or higher, and typically at least 98%. ACPP-2/9 was labeled with ^177^Lu as previously described [1].

#### 3.4.2. Cell Culture

HT-1080 fibrosarcoma cells acquired from the American Type Culture Collection (ATCC) were maintained under standard culture conditions in Eagle’s Minimal Essential Medium (MEM) supplemented with 10% heat inactivated fetal bovine serum (Gibco, Waltham, MA, USA), penicillin (100 U/mL), streptomycin (100 µg/mL), and 2 mM Glutamax (Gibco).

#### 3.4.3. *In Vitro* Cell Incubation with Radiolabeled ACPP Probes

^177^Lu-ACPP-B (50 µM) was activated prior to cell incubation with recombinant human MT1-MMP (100 nM) for 2 h in 50 mM Tris, 200 mM NaCl, 10 mM CaCl_2_ and 10 µM ZnCl_2_ at pH 7.5, as was assessed by RP-HPLC. HT-1080 cells were cultured in 12-well plates. At 95% confluency, cells were washed with Dulbecco’s PBS (Gibco) and subsequently incubated in triplicate with 1.25 µM ^177^Lu-non-ACPP, 1.25 µM uncleaved ^177^Lu-ACPP-B, 1.25 µM pre-cleaved ^177^Lu-ACPP-B, and 1.25 µM ^177^Lu-CPP-B (0.25 MBq ^177^Lu) respectively in serum-free medium (1 mL). After 3 h of incubation, cells were washed 3× with Dulbecco’s PBS, and treated with trypsin (0.25% *w/v* trypsin-EDTA (Gibco)) to digest and remove surface-bound peptide [32] and harvest the cells. The trypsin activity was inhibited by addition of MEM, after which the cells were isolated by centrifugation (1000 rpm, 5 min, RT). Cell pellets and all wash fractions were analyzed for ^177^Lu radioactivity (115–270 keV) by a γ-counter (Wizard 1480, PerkinElmer).

*MMP blocking study:* HT-1080 cells were cultured in 12-well plates. At 95% confluency, cells were washed with Dulbecco’s PBS (Gibco) and subsequently incubated (*n *= 4) with 1.25 µM ^177^Lu-non-ACPP, 1.25 µM ^177^Lu-ACPP-B, and 1.25 µM pre-cleaved ^177^Lu-ACPP-B + 50 µM GM6001, respectively in serum-free medium (1 mL). After 4 h of incubation, cells were washed 3× with Dulbecco’s PBS and harvested by trypsination (0.25% *w/v* trypsin-EDTA (Gibco)). The trypsin activity was inhibited by addition of MEM, after which the cells were isolated by centrifugation (1000 rpm, 5 min, RT). Cell pellets and all wash fractions were analyzed for ^177^Lu radioactivity (115–270 keV) by a γ-counter.

#### 3.4.4. Animal Studies

All animal procedures were approved by the ethical review committee of the Maastricht University Hospital (The Netherlands), and were performed according to the principles of laboratory animal care (NIH publication 85–23, revised 1985) [33], and the Dutch national law “Wet op Dierproeven” (Stb 1985, 336). Male Swiss mice (body weight >25 g, Charles River Labs) were housed in an enriched environment under standard conditions: 21–23 °C, 50%–60% humidity, and 12 h-lightdark-cycles for >1 week. Food and water were freely available.

#### 3.4.5. Mouse Model of Myocardial Infarction (MI)

MI was induced by permanent ligation of the left anterior descending coronary artery (LAD) using published procedures [34]. In short, animals were subcutaneously injected with buprenorphine (0.1 mg/kg) and 30 min later anesthetized with isoflurane. Animals were intubated and ventilated with 100% oxygen with a rodent respirator. After left thoracotomy between ribs four and five, the LAD was ligated with a 6–0 prolene suture. The chest and skin were closed with 5–0 silk sutures. The animal’s temperature was continuously measured rectally and maintained at 36.5–37.5 °C during surgery. After surgery, animals were allowed to recover at 30 °C overnight.

#### 3.4.6. Blood Kinetics and Biodistribution

Biodistribution experiments were performed on MI-mice (*n =* 5 per probe) by i.v.-injection of ^177^Lu-ACPP-B (10 nmol/100 µL, *ca.* 4.0 MBq ^177^Lu), or ^177^Lu-CPP-B (10 nmol/100 µL, *ca.* 4.0 MBq ^177^Lu). For animals injected with ^177^Lu-ACPP-B, blood samples were withdrawn from the vena saphena at selected time points (3, 9, 27, and 81 min), weighed, and diluted to 1 mL with MilliQ water. The mice were anesthetized with isoflurane 5 h after i.v.-injection, subjected to 2% (*w/v*) Evans Blue i.v.-injection (50 mg/kg) and sacrificed 2 min later by cervical dislocation. Organs and tissues of interest were harvested and weighed. The hearts were cooled to 4 °C, cut in 1 mm slices from apex to base. Infarct, border, and remote areas were isolated based on Evans Blue staining. The ^177^Lu radioactivity of all samples was measured in a γ-counter (Wizard 1480; PerkinElmer) along with standards to determine the injected dose per gram (%ID/g). The energy window was set to 155–380 keV. The average probe concentration at time 0 (C_0_) was calculated by fitting the blood clearance data to the 2-phase exponential decay function Y = span_1_ × exp(−K_1_ × X) + span_2_ × exp(−K_2_ × X) + Plateau using GraphPad Prism. The area under the curve (AUC) was determined and the blood half-life, the time point at which the AUC reaches 50% of the total AUC, was subsequently derived using MatLab. The average volume of distribution was calculated using the formula V_D_ = dose/(mean body weight × C_0_) [L/kg].

## 4. Conclusions

We have successfully developed a series of activatable cell penetrating peptides (ACPP) sensitive to MT1-MMP. The most potent ACPP analog, ACPP-B, was selected by MT1-MMP sensitivity and enzyme specificity assays. In *in vitro* cell assays, radiolabeled ACPP-B showed efficient cellular uptake upon activation. Furthermore, the level of *in vivo* background activation in the vasculature was decreased compared to ACPP-2/9, an earlier well-studied MMP-2/9 ACPP probe, while an increased uptake in infarcted heart tissue was observed compared to remote heart tissue, which warrants future research into the *in vivo* biodistribution and behavior of ACPP-B.

## Supplementary Materials

Supplementary materials can be accessed at: http://www.mdpi.com/1420-3049/20/07/12076/s1.
