# Supplementary material for: Development of Radiolabeled Membrane Type-1 Matrix Metalloproteinase Activatable Cell Penetrating Peptide Imaging Probes
Source: Molecules. 2015 Jul 2;20(7):12076–92. doi: 10.3390/molecules200712076 (PMC6332093; doi:10.3390/molecules200712076)
Supplement: Supplementary file 1 [file molecules-20-12076-s001.pdf]

Supplemental Information

Supplementary Discussion: Design Consideration ACPP-C

Initially ACPP-C was synthesized on the solid phase with a tyrosine residue at the N-terminus. Incubation of this peptide in PBS in a Pierce iodination tube (Thermo Scientific) resulted in oxidation of the C_{mob} residue as was indicated by LC-MS (+16 Da, and +2*16 Da adducts were observed (Supplementary Figure S7). To prevent oxidation of C_{mob}, ^{125}I was intended to be introduced via an ^{125}I-labeled SHPP-*N*-hydroxysuccinimide ester residue. We initially tested ACPP-C without SHPP for MT1-MMP sensitivity, while ACPP-C with a non-radioactive SHPP moiety was used for the enzyme specificity assay.

Supplementary Material and Methods: Iodination Strategies ACPP-B

Direct labeling in iodination tube: ^{125}I (PerkinElmer) in PBS (6.0 µL, 11.5 MBq) was mixed with ACPP-B in MilliQ water (10.4 µL, 45 nmol), and PBS (433.6 µL) in an Iodogen iodination tube (Pierce) for 20 min, at 600 rpm and 23 °C, and transferred to a siliconized 1.5 mL tube. The ^{125}I labeling yield was determined by radio-TLC, using iTLC-SG strips eluted with 20 mM citric acid at pH 5.2. A >95% radiochemical purity was observed. As control, ACPP-B in MilliQ water (10.4 µL, 45 nmol) was mixed with PBS (433.6 µL) in an Iodogen iodination tube for 5 min, at 600 rpm and 23 °C, followed by LC-MS analysis. Found mass: 9197.7 Da, Calc. MW: 9197.8 Da for ACPP-B peptide dimer.

Indirect labeling: ^{125}I (PerkinElmer) in PBS (439.6 µL, 11.5 MBq) was activated in an Iodogen iodination tube for 7 min according to vendor's protocol, and transferred to a siliconized 1.5 mL tube containing ACPP-B in MilliQ water (10.4 µL, 45 nmol) and mixed for 20 min. As control PBS incubated in an Iodogen iodination tube for 7 min was transferred to a siliconized 1.5 mL tube containing ACPP-B in MilliQ water (10.4 µL, 45 nmol), and mixed for 20 min, followed by LC-MS analysis. Found mass: 4597.3 Da, Calc. mass: 4597.3 Da for ACPP-B.

For this indirect labeling procedure, a ~60% labeling yield was typically observed by radio-TLC as described before. Impure ^{125}I-ACPP-B, mixed with 500 uL 0.1% TFA in MilliQ, was purified by solid phase extraction on a light C_8 Sep-Pak cartridge (Waters). Unfortunately, a >95% radiochemical purity could not be achieved. In detail, the cartridge was washed with 5 × 1 mL 0.1% TFA in MilliQ after peptide loading. Subsequently, the cartridge was washed with 1 mL 20% EtOH in MilliQ, 1 mL 30% EtOH in MilliQ, 1 mL 50% EtOH in MilliQ, 1 mL 70% EtOH in MilliQ, and 1 mL 96% EtOH in MilliQ. All solutions contained 0.1% TFA. Radioactivity (in MBq) of all fractions was determined, followed by radiochemical purity analysis of fractions of interest using radio-TLC.

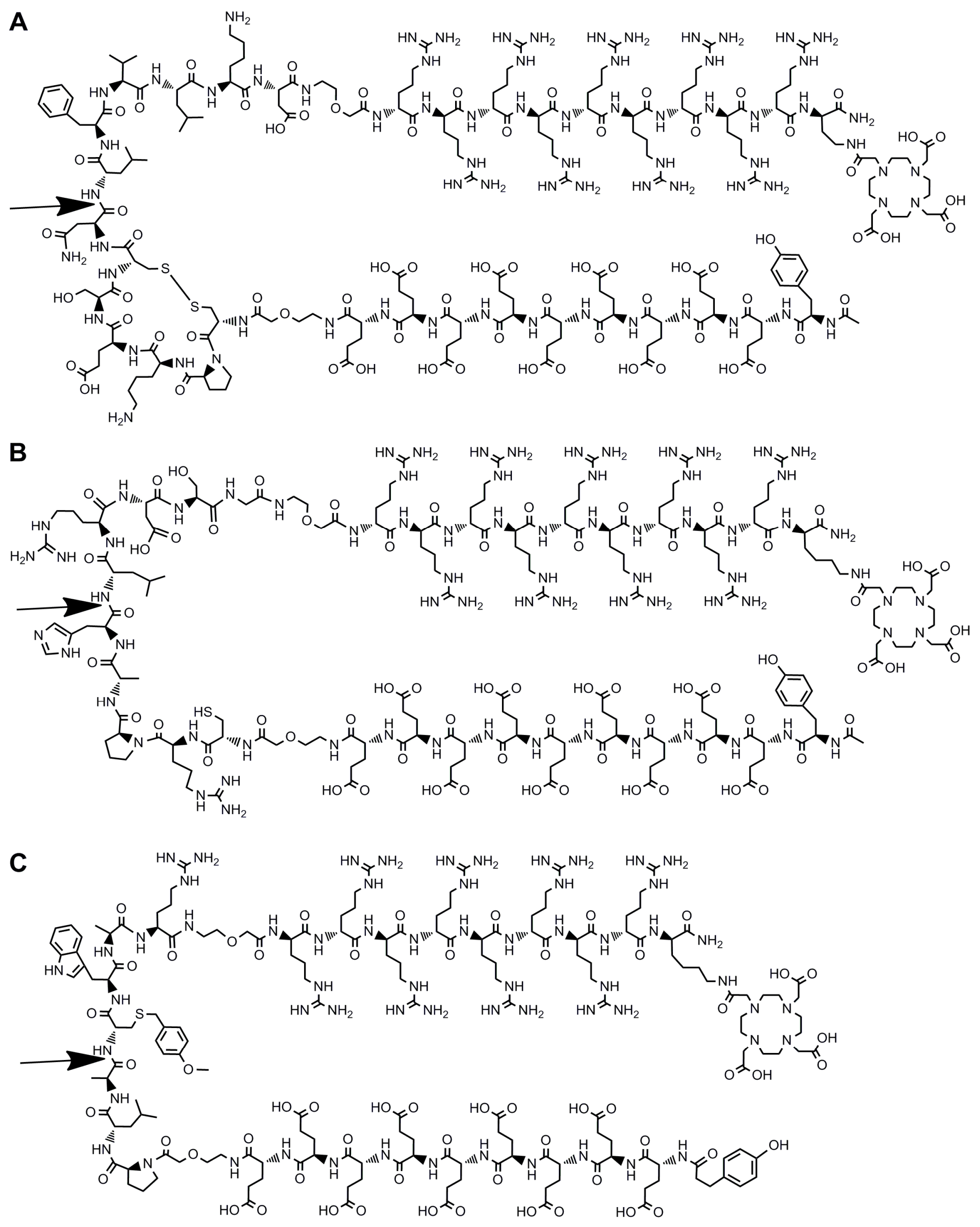

Figure S1. Molecular structure of (**A**) Ac-y-e_9-X-C*PKESC*NLFVLKD-X-r_9-dab(DOTA)-NH_2 (ACPP-A), (**B**) Ac-y-e_9-X-CRPAHLRDSG-X-r_9-k(DOTA)-NH_2 (ACPP-B), and (**C**) SHPP-e_9-X-PLAC_{mob}WAR-X-r_8-k(DOTA)-NH_2 (ACPP-C). The arrows indicate the MT1-MMP cleavage sites. In ACPP-A, the two cysteines, indicated by C*, form an intramolecular disulfide bridge. X represents 3-oxapentanoic acid (O1Pen).

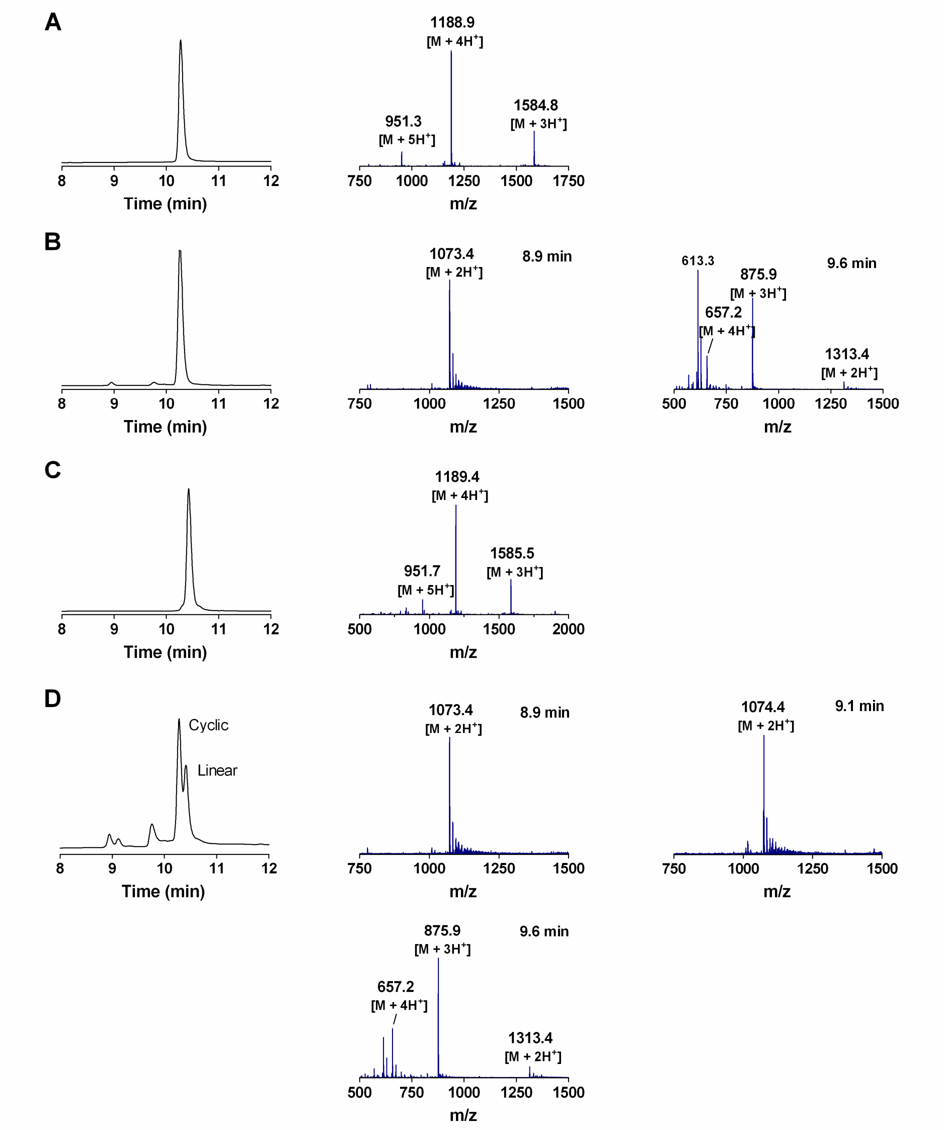

Figure S2. LC-MS characterization of cyclic ACPP-A analog Ac-y-e_9-C^*PKESC*NLFVLKD-r_9-dab(DOTA)-NH_2 and linear ACPP-A analog Ac-y-e_9-CPKESCNLFVLKD-r_9-dab(DOTA)-NH_2 (0.1 mM) (**A** & **C**) before and (**B** & **D**) after 19h incubation with MT1-MMP (0.13 μM), respectively. The left and right graphs show the UV absorbance chromatogram and the mass spectra, respectively. C^* represents cysteine residues that are linked via an intramolecular disulfide bridge. (**A**) MS spectrum of Ac-y-e_9-C^*PKESC*NLFVLKD-r_9-dab(DOTA)-NH_2, obsd. 4749.4 Da, calcd. 4749.3 Da. (**B**) MS spectra at 8.9 min (Neutralizing domain, obsd. 2143.8 Da, calcd. 2143.7 Da for Ac-y-e_9-C^*PKESC*N-COOH), and at 9.6 min (CPP, obsd. 2623.7 Da, calcd. 2623.7 Da for H_2N-LFVLKD-r_9-dab(DOTA)-NH_2). (**C**) MS spectrum of Ac-y-e_9-CPKESCNLFVLKD-r_9-dab(DOTA)-NH_2, obsd. 4751.4 Da, calcd. 4751.4 Da. (**D**) MS spectra at 8.9 min (cyclic neutralizing domain, obsd. 2143.8 Da, calcd. 2143.7 Da for Ac-y-e_9-C^*PKESC*N-COOH), at 9.1 min (linear neutralizing domain, obsd. 2145.8 Da, calcd. 2145.8 Da for Ac-y-e_9-CPKESCN-COOH), and at 9.6 min (CPP, obsd. 2623.7 Da, calcd. 2623.7 Da for H_2N-LFVLKD-r_9-dab(DOTA)-NH_2).

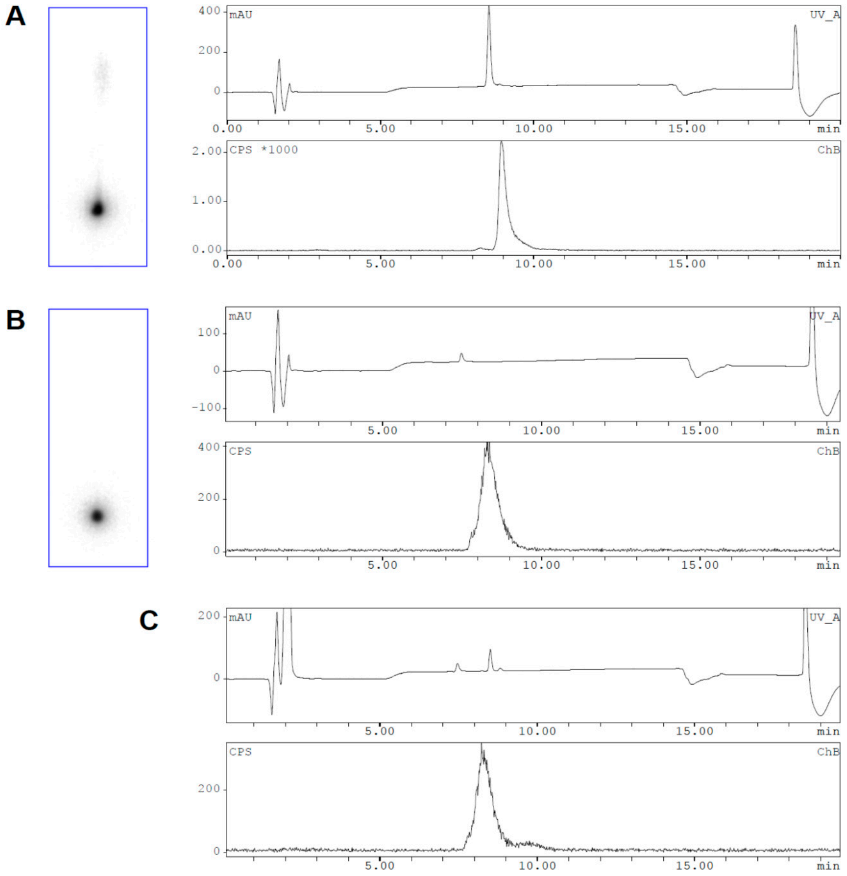

Figure S3. iTLC and γ-HPLC spectra of (**A**) uncleaved ^{177}Lu-ACPP-B and (**B**) ^{177}Lu-CPP-B. (**C**) γ-HPLC spectrum of pre-activated ^{177}Lu-ACPP-B, showing >95% cleavage. Radiochemical purities were >99% as assessed by iTLC. The upper panel shows the UV absorbance chromatogram at 212 nm and the lower panel shows the γ-radiation monitored by a γ-detector.

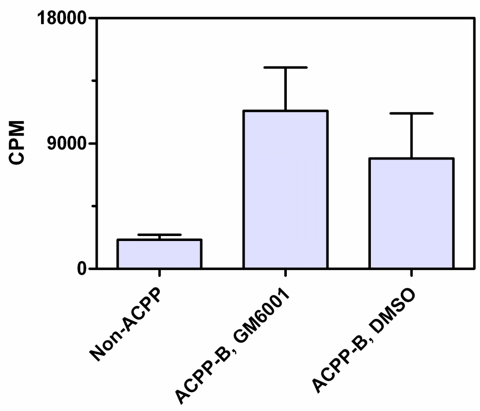

Figure S4. Cellular uptake of ^{177}Lu-non-ACPP, ^{177}Lu-ACPP-B in the presence of 50 µM GM6001 (from 50 mM stock in DMSO), and ^{177}Lu-ACPP-B in the absence of GM6001 after 3h incubation with HT-1080 cells, assessed by γ-counting. Final concentration of DMSO was 0.1% v/v for both ACPP-B groups. Data are presented as mean ± SD.

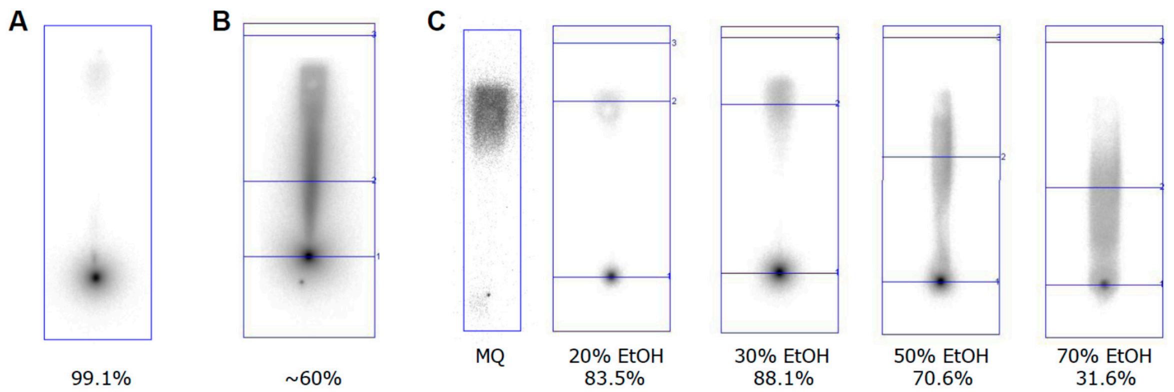

Figure S5. Radio-TLC analysis of ^{125}I iodination of ACPP-B. (**A**) Direct iodination of ACPP-B in an iodination tube results in >95% radiochemical purity, but also in ACPP-B peptide dimer formation. (**B**) Prior activation of ^{125}I and subsequent iodination in an siliconized tube resulted in ~60% labeling yields. (**C**) iTLC results for solid phase extraction fractions of unpure ACPP-B. Maximum obtained purity was <90%.

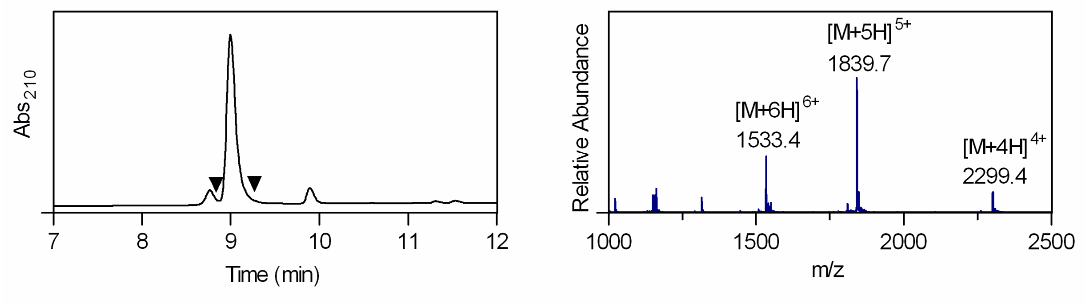

Figure S6. LC-MS characterization of ACPP-B incubated in PBS in an iodination tube for 5 min in the absence of ^{125}I. The left and right graphs show the UV absorbance chromatogram and the mass spectrum of the UV-peak bracketed by the arrowheads, respectively. Observed mass of 9194 Da corresponded to the calculated mass of an ACPP-B peptide dimer linked to each other via a intermolecular disulfide bridge (calc. mass 9193 Da).

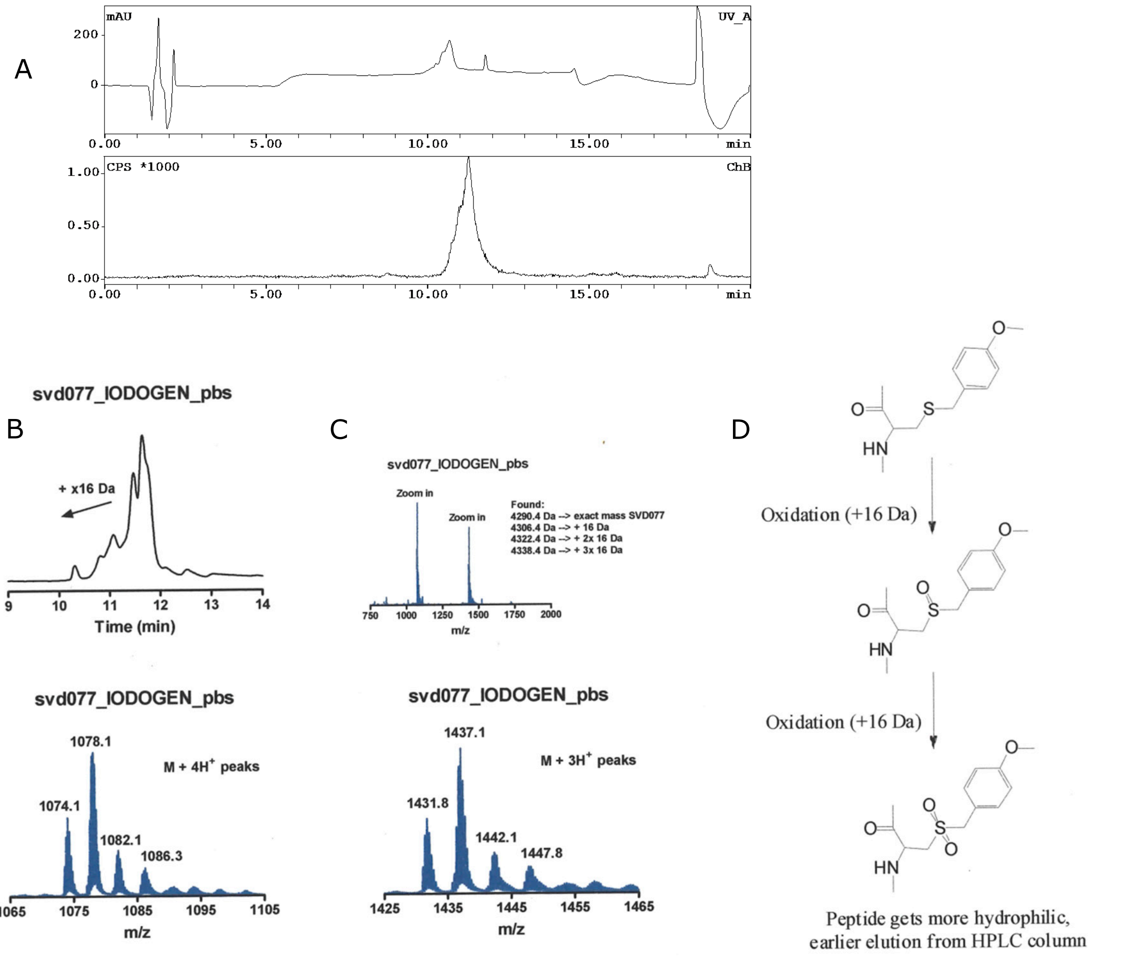

Figure S7. (**A**) γ-HPLC spectra of ACPP-C analog y-e$_9$-x-PLAC$_{mob}$WAR-x-r$_8$-k(DOTA)-NH$_2$ labeled with ^{125}I in an iodination tube for 50 min, 500 rpm, RT. The upper panel shows the UV absorbance chromatogram at 212 nm and the lower panel shows the γ-radiation monitored by a γ-detector. The panels show shoulder peaks indicative for a non-homogeneous product. (**B**) LC-MS characterization of the same peptide (encoded as svd077) incubated in an iodination tube in PBS for 50 min, 500 rpm, RT in the absence of ^{125}I. The UV absorbance chromatogram (left) and the mass spectrum (right) of the UV-peak (11.2–12.0 min) are shown. (**C**) Zoomed-in mass spectra for the 4+ (left) and 3+ (right) ion set. Observed masses: 4290.4 Da (y-e$_9$-X-PLAC$_{mob}$WAR-X-r$_8$-k(DOTA)-NH$_2$, calc. mass 4290.4 Da), 4306.4 Da (y-e$_9$-X-PLAC$_{mob}$WAR-X-r$_8$-k(DOTA)-NH$_2$ + 16 Da), and 4322.4 Da (y-e$_9$-X-PLAC$_{mob}$WAR-X-r$_8$-k(DOTA)-NH$_2$ + 2 × 16 Da). (**D**) Schematic representation of oxidation of C$_{mob}$ amino acid. x represents amino-hexanoic acid.

Table S1. Biodistribution results of 10 nmol ^{177}Lu-CPP-B 5h post-injection in MI-mice. The data are mean %ID/g ± SD. ^{177}Lu-CPP-B uptake in infarct was significantly higher in infarct compared to remote ($p < 0.01$).

	**^{177}Lu-CPP-B (*n* = 5)**
Blood	0.14 ± 0.06
Heart, infarct	1.69 ± 0.50
Heart, remote	0.32 ± 0.11
Muscle	0.10 ± 0.02
Lung	0.67 ± 0.25
Spleen	4.17 ± 0.82
Liver	33.2 ± 3.98
Kidney	10.1 ± 1.61
Fat	0.16 ± 0.07
Thigh bone	2.61 ± 0.25
Brain	0.01 ± 0.01
